# Test-retest measurements and digital validation for *in vivo* neuroscience

**DOI:** 10.1038/sdata.2014.57

**Published:** 2015-01-20

**Authors:** Franco Pestilli

**Affiliations:** 1 Department of Psychological and Brain Sciences, Programs in Neuroscience and Cognitive Science, Indiana University, 1101 E 10th Street, Bloomington, Indiana 47405, USA

**Keywords:** Neuroscience, Neuroinformatics, Brain imaging

Neuroscience is transforming. Brain data collected in multitudes of individuals and institutions around the world are being openly shared, moved from office desks and personal storage devices to institutionally supported cloud systems and public repositories—effectively bringing Neuroscience into the era of Big Data. This is an important evolution in Neuroscience, since the value of open data sharing has not always been recognized^1–5^. Indeed, the value of neuroscience data sharing was highlighted in a recent Special Focus issue in *Nature Neuroscience*^6^. As a leading example of this trend, this month *Scientific Data* is launching a collection of articles presenting diverse brain imaging data sets, which collectively provide valuable resources for validation and reproduction of neuroscience findings based on human magnetic resonance imaging (hMRI).

hMRI stands out from the neuroscientific crowd because standards have begun to be established for hardware, file formats (for example, see DICOM, http://dicom.nema.org/ and NIfTI, http://nifti.nimh.nih.gov/) and acquisition protocols^7–9^. Many hopes are also being put on large-scale processing of big neuroimaging data and the potential impact it will have on understanding the human brain. But, while there is clear value in data standardization, there is also value in collecting and sharing heterogeneous datasets generated from a variety of hardware and acquisition protocols. This is precisely the strength of the datasets published within the *Scientific Data* MRI Reproducibility Collection.

The datasets in the Collection provide unique hMRI measurements collected using different modalities, including both functional and structural magnetic resonance imaging as well as associated physiological and behavioral data. Central to this Collection are data aggregated and released by the Consortium for Reliability and Reproducibility (CoRR), which has grown out of the established 1000 Functional Connectomes Project^10^. The CoRR measurements were repeated at least twice providing the foundations for test-retest reliability estimates that can be used in combination with modern statistical methods to validate and reproduce scientific results. The manuscript by Zuo *et al*.^11^ provides an overview of the CoRR project, and describes data collected by 36 research groups in 18 institutions and three continents (America, Asia and Europe). Also in association with CoRR, Gorgolewski *et al*.^12^ report functional imaging data acquired with spatial resolutions higher than current standards (1.5 and 0.75 mm^3^). Data were collected repeatedly in the same individuals and accompanied by physiological measurements (such as subjects’ respiration and heartbeat) and cognitive and affective measures. Overall, the large sample size, rich diversity and the repeated measurements across the CoRR datasets provide the multiplicity and scale necessary for building, evaluating and testing models of human brain functional connectivity. Also included in the launch of this Collection, Maclaren *et al*.^13^ collected human brain anatomy data using the standard protocol established by the Alzheimers’ Disease Neuroimaging Initiative (ADNI)^14^. Importantly, data acquisition was repeated multiple times in each individual brain providing first hand resources for test-retest reliability estimates on the protocol. Additional articles describing valuable brain imaging datasets will be added to this Collection in a rolling manner.

Scientific enterprises are limited by the nature of the available data; the signal and noise in the data limit understanding. For this reason, scientists strive to measure new signals and collect better data to provide new scientific insights or validate previous findings. Neuroscience has traditionally stood on the relatively strong shoulders of postmortem tissue measurement. Such methods are still often accepted as the gold standard for anatomical measurements of the brain and form the basis of numerous theoretical accounts of brain function. But modern measurement technologies are bringing strong value to digital, *in vivo* neuroscience, because it can chart the relation between human brain anatomy, brain function, and behavior within the same individual. These technologies allow repeated measurements in the same individual as well as in large human populations. Digitally stored data can be easily shared and accessed. Measurements can be replicated in new populations and results can be challenged or replicated by new datasets. Large, high-resolution, heterogeneous data resources like those presented in *Scientific Data* MRI Reproducibility Collection, will be particularly valuable to investigators working in the modern era of digital neuroscience.

Modern digital neuroscience necessitates new methods for results evaluation and validation. These methods can use the signal and noise in the data to distinguish true neurobiological features from artifactual ones. In recent work^15^, we have developed a method called LiFE (Linear Fascicle Evaluation; https://francopestilli.github.io/life/) to evaluate the accuracy of brain connectomes^16^ built using diffusion imaging (dMRI) and computational tractography. The method, briefly summarized in Fig. 1, uses a forward model approach and repeated measurements similar to the ones in the *Scientific Data* MRI Reproducibility Collection. First, a model of brain connections is built from one dataset using available tractography algorithms. The model is then evaluated by measuring how well it predicts a second, independent dataset (cross-validation). The idea here is that a neuroscience model should predict the signal but not the noise in the data. So, when built on test data, the model can be validated by measuring how well it predicts retest data—data that contain presumably similar signal, but different noise. If the model predicts both test and retest datasets well, it can be interpreted as accurate, as it captures the relevant signal without bias from the noise. However, a bad model built on the test dataset may capture both the signal and noise in the test data, and will inaccurately predict the retest data, which contains different noise. This process is limited only by the ‘signal’ information content in the data. Other approaches to validation compare estimates from in-vivo neuroimaging data to independent postmortem or synthetic data (see ref. 15 for a discussion) but these cannot be applied routinely to living brains, nor correlated to human health and behavior. We have used LiFE and digital-validation to identify the anatomy of a major human brain fascicle missed by postmortem measurements (refs 15,17, Fig. 1c) and to improve our understanding of the network of human white matter fascicles supporting face and place processing^18^, providing an example of how data such as that published in the *Scientific Data* MRI Reproducibility Collection, in combination with modern statistics, can benefit the neuroscience community.

Whereas the value of open data sharing is clear, advances are still needed to support wider sharing—one being better ways to publish data. Investigators collecting data, those interested in using the data and their funding bodies, all benefit from data publishing. Among the many benefits of data publication are credits for authors, the review process and data availability^19^. Published data become searchable using standard mechanisms of scientific referencing (for example, PubMed and Google Scholar). Published data can be naturally cross-linked with research articles, potentially increasing both neuroscientific understanding and reproduction of results.

The US National Institutes of Health has recently issued a funding initiative focused on cooperative neuroimaging efforts between investigators in Neuroscience and Clinical Sciences (https://grants.nih.gov/grants/guide/pa-files/PAR-14-281.html), asking that data be collected using the same protocols developed for the Human Connectome Project (HCP) (ref. 8). The NIH program is an excellent opportunity for enforcing new standards for quality of data collection initiated by the HCP, with benefits for both clinical and basic researchers. These data will be made public and add to the growing body of efforts collecting neuroscientific data. Along the same lines the UK Medical Research Council is promoting as part of its 2014–2019 strategic plan both the collection of large genetic and neuroimaging phenotypic datasets to understand predisposition to disease, as well as the creation of a common platform for dementia research within the UK (http://www.mrc.ac.uk/news-events/publications/strategic-plan-2014-19/).

Another project of particular note, the BRAIN initiative (http://www.braininitiative.nih.gov/2025/), is promoting the collection of yottabytes (10^24^) of data^20^. Analyzing these data effectively will require large computers and fast computing methods. Because moving such large datasets will be prohibitive, software and computations will have to move to computers physically close to the stored data. This is the current model in Big Data analytics where distributed data are analyzed by software deployment without moving data (see for example, Spark and Hadoop). Dataset size will likely require changes to the common centralized database architectures, promoting distributed or ‘federated’ models. Federated databases stored at multiple institutions, and facilitated by modern computing tools, could provide rich datasets with heterogeneous multimodal measurements that investigators will access publicly or as part of research consortia by deploying computational software. Furthermore, increased standards for reproducibility of neuroscience results will require data and the computations applied to them to be tracked and linked together permanently. Computing environments that can implement this properly are already emergin^21^, (see for example, IPython, http://ipython.org/ and Docker, https://www.docker.com/), and may become particularly useful when combined with concepts from highly parallel computing. A federated database for neuroscience (NIMS, Neurobiological Image Management System, https://scitran.stanford.edu/nims/) is currently being developed as part of the Stanford Project on Scientific Transparency using precisely these technologies.

Finally, the next challenge for Big Data Neuroscience will be capturing the human behavior associated with brain measurements. Standards for sharing brain data are already being developed, but standards for behavioral data sharing will be more challenging because consensus about file formats, software for data collection and tasks remain elusive. This is a serious challenge for the whole enterprise, as it has been noted that, ‘nothing in neuroscience makes sense except in the light of behavior’^22^, reported in ref. 23. The future of neuroscience will comprise coordinated efforts for collecting multiple, standardized and heterogeneous datasets, as well as developing analytic tools for understanding them. This task will require dedicated funding schemes for training and sustaining new generations of investigators with skillsets to lead these efforts^23^.

## Figures and Tables

**Figure 1:**
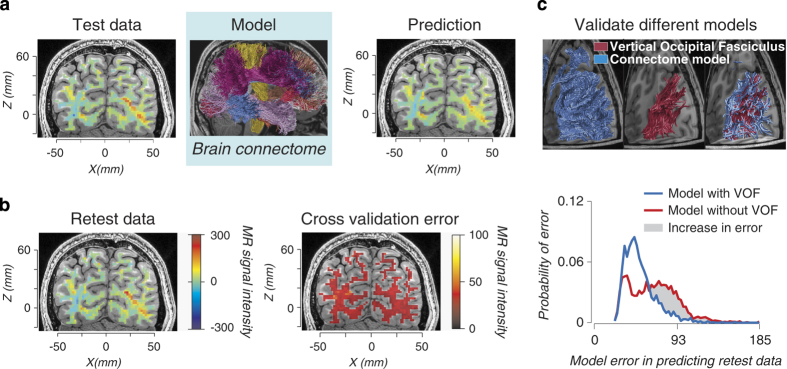
Linear Fascicle Evaluation (LiFE) an example of digital validation for human connectomes. Diffusion-weighted magnetic resonance imaging (dMRI) and fiber tractography allow *in vivo* mapping of human white-matter fascicles and connections. Tractography takes diffusion measurements as input and produces a large collection of white-matter fascicles as output, the connectome. The LiFE method evaluates the evidence in favor of different connectomes. LiFE takes full connectomes as inputs and predicts diffusion measurements as output. The difference between the measured and predicted diffusion is used to compute the connectome prediction error and the evidence in favor of a connectome. The prediction error is also used to evaluate the evidence supporting properties of the connectome such as the evidence for a tract or connection. (**a**) Building a forward model of diffusion data from a human connectome. Test data: a map of measured diffusion MRI modulation is presented in a typical coronal brain slice and for a single diffusion direction, left panel. Model: A whole brain connectome is estimated using fiber tractography and the Test data, middle panel. Prediction: LiFE uses the individual fascicles in the connectome to generate the predicted diffusion modulation in the same brain. Fascicles not contributing successfully to the Prediction are eliminated, right panel. (**b**) Evaluating the connectome model using the retest data and cross validation. Retest data: A map of a second measurement of diffusion MRI modulation made in the same individual brain and slice. The noise in the data introduces differences in the measured diffusion modulation, left panel. Visually compare Test and Retest data to appreciate the difference. Cross validation error: The Prediction and the Retest data are compared to evaluate the accuracy of the model, right panel. (**c**) Evaluating the evidence for the vertical occipital fasciculus. The neighborhood of fascicles belonging to the posterior portion of the connectome is overlaid on brain slices, left top panel, blue. The portion of the vertical occipital fasciculus (VOF) is identified in red, top middle panel. The VOF is shown with the rest of connectome fascicles passing through the same white matter region, right top panel, red and blue. To test the evidence in favor of the VOF, the fascicles in red are removed from the connectome model and the cross-validated prediction error is compared for the full connectome model (red and blue fascicles) and for the model without the VOF (blue fascicles alone). The cross-validated prediction error increases when the VOF is removed, bottom panel red, this indicates that the data offer evidence for the VOF. Portions of this figure have been adapted from Fig. 2 in ref. 15.
